# Management for lymphatic malformations of head and neck

**DOI:** 10.3389/fneur.2024.1450102

**Published:** 2024-10-02

**Authors:** Weijia Yang, Huaijie Wang, Chong Xie, Weilong Lin, Peihua Wang, Zhengtuan Guo

**Affiliations:** Department of Pediatric Surgery and Vascular Anomalies, Xi’an International Medical Center Hospital, Xi’an, China

**Keywords:** lymphatic malformations, neck, macroglossia, sclerotherapy, surgery, tracheotomy

## Abstract

**Background:**

To explore the management of lymphatic malformation in head and neck.

**Methods:**

This is a retrospective study at a single center. Data on demographic, surgery, sclerotherapy and follow-up information were collected from our Vascular Anomalies Center database. Patients with lymphatic malformation of head and neck who had undergone surgery and sclerotherapy between March 2020 and March 2024 were included.

**Results:**

There were 94 patients in this study, the lesion sites included head (*n* = 60), tongue (*n* = 7), neck (*n* = 41), pharynx (*n* = 7), and head and neck (*n* = 7). Symptoms included bleeding (*n* = 6), infection (*n* = 2), dyspnea (*n* = 2), dysphonia (*n* = 4), and dysphagia (*n* = 4). Lymphatic malformation included macrocystic (*n* = 61), microcystic (*n* = 12) and mixed (*n* = 21). Surgeries for LM included radical resection, subtotal or partial resection and staged surgeries. Sclerotherapies included bleomycin monotherapy and combined sclerotherapy with ethanol and bleomycin, under ultrasound or fluoroscopy guidance. The follow-up period was from 3 months to 1 year. The therapeutic effect was evaluated according to the size of the treatment area. 55 patients, 21 patients, 11 patients and 7 patients were evaluated with excellent, good, moderate and no response, respectively.

**Conclusion:**

Surgical resection, sclerotherapy and the combination of the two are efficacious treatment modalities for head and neck LM. Combined with oral drugs and other new therapies may be warranted in future for challenging conditions.

## Introduction

Lymphatic malformations (LM) are classified as a low-flow vascular malformation, which belongs to benign lesions, but its harm and treatment are related to the morphologic type, anatomic location and extent. Lymphatic malformations are divided into 3 types (1), macrocystic, microcystic and mixed cystic, which are lymphatic masses composed of cysts of varying sizes. LM are common in the head and neck, accounting for about 75 percent (2). The prognosis is worse when the lesion involves tongue, floor of mouth and pharynx (3).

Currently, management include conservative observation, surgery, sclerotherapy and oral medication. This paper reviewed the treatment of LM in head and neck at our center.

## Material and method

A 3-year (March 2020 to March 2024) retrospective electronic chart review of all patients with LM admitted to Xi’an International Medical Center Hospital was performed. Keywords lymphatic malformation was used to identify patients in our electronic medical records system. Diagnosis was based on clinical history, photographs, physical examination, color Doppler ultrasound, and magnetic resonance imaging (MRI). Approval was obtained from Xi’an International Medical Center Hospital Institutional Review Board. Color Doppler ultrasound and MRI images were reviewed for morphological subtype of the LM, tissue involvement, therapeutic response evaluation.

### Surgical resection

In patients with macrocystic LM, pre-sclerotherapy treatment of LM involving the airway may require tracheostomy, as the post-procedure swelling may cause airway obstruction. A preemptive tracheostomy may be required. For microcystic LM, the therapeutic response of sclerotherapy was often limited, and surgical debulking is needed, such as macroglossia, LM involving glottis and epiglottis. However, cosmetic concern following facial lesion surgery needs to be weighting. For mixed LM, surgery was considered if response was poor to sclerotherapy. Additional sclerotherapy for residua via drainage tube or intraoperative injection could be required in some cases.

Surgeries for LM included radical resection, subtotal or partial resection and staged surgeries. During some surgeries, the intercapsular separation can be removed to form a large cavity, creating conditions for intraoperative or postoperative sclerotherapy.

### Sclerotherapy

All the children underwent preoperative routine examination after admission, and interventional therapy was performed after no contraindications were confirmed. Before operation, the lesions were marked on the body surface according to the imaging examination. Before puncture, bleomycin was dissolved in iodohexol contrast medium, and the concentration was between 0.5 mg/mL and 1 mg/mL according to the depth of the puncture site. A maximum of 0.5 mg/kg bleomycin was used per session. The puncture is under the guidance of ultrasound. Bleomycin alone was only used for treatment of microcystic or mixed LM (no obvious lymph fluid or a small amount of lymph fluid was extracted after puncture). Bleomycin was dissolved in contrast medium, and any lymphatic fluid (LM intracavity or suspected intracavity) was extracted from the lesion by puncture into the lesion, and the drug should be injected, mainly into the lesion intracavity and interstitial. According to the skin markings before surgery, multi-point injection can be performed to manage localized lesion. Postprocedural pressure bandage could help local adhesion and closing the capsule cavity. The response was evaluated 3 months after discharge, repeated injection may be needed. For macrocystic or mixed LM that can extract lymph fluid by puncture, rinse with anhydrous ethanol. If the cyst was large, double-needle technique could be used to reduce the risk of anhydrous ethanol exosmosis related necrosis. Then the mixture of contrast and bleomycin was injected. Multi-sites injection also can be used to maximize the sclerosant diffusing in the lesion area.

Clinical evaluation scale (4) was used to evaluate the treatment effect of patients with or without related symptoms and signs: mass regression ≥90%, no symptoms and signs (excellent); mass regression ≥50%, no obvious symptoms and signs (good); mass regression <50%, occasionally associated symptoms and signs (moderate); The mass did not significantly subside, and the symptoms and signs were not significantly improved; or larger mass or worse symptoms and signs than before treatment (no response). We used three senior physicians to independently evaluate follow-up patients based on Doppler ultrasound, MRI and appearance. If at least two physicians are the same rating, the patient is considered to have this rating.

## Result

There were 94 patients in the retrospective study (Table 1), the lesions included head (*n* = 60), tongue (*n* = 7), neck (*n* = 41), pharynx (*n* = 7) and head and neck (*n* = 7). Symptoms included bleeding (*n* = 6), infection (*n* = 2), dyspnea (*n* = 2), dysphonia (*n* = 4), and dysphagia (*n* = 4). Morphological subtype included macrocystic (*n* = 61), microcystic (*n* = 12) and mixed (*n* = 21).

**Table 1:** Clinical characteristics of the study population.

Variable	Sclerotherapy (*N* = 84)	Surgical resection (*N* = 10)	Total (*N* = 94)
Sex (No.)			
Female	36	3	39
Male	48	7	55
Age (y)			
Median	2	2	2
Range	1–11	1–5	1–11
Location			
Head	52	8	60
Tongue	4	3	7
Neck	37	4	41
Pharyngeal	5	2	7
Head and Neck	5	2	7
Symptom			
Hemorrhage	5	1	6
Infection	2	0	2
Dyspnea	0	2	2
Cacoepy	1	3	4
Dysphagia	1	3	4
Morphological subtype			
Macrocystic	61	0	61
Microcystic	5	7	12
Mixed	18	3	21
Operative duration (min.)			
Median	35	87	38
Range	5–63	57–156	5–156
Follow-up evaluation			
Excellent	52	3	55
Good	18	3	21
Moderate	8	3	11
No response	6	1	7

**Figure 1:**
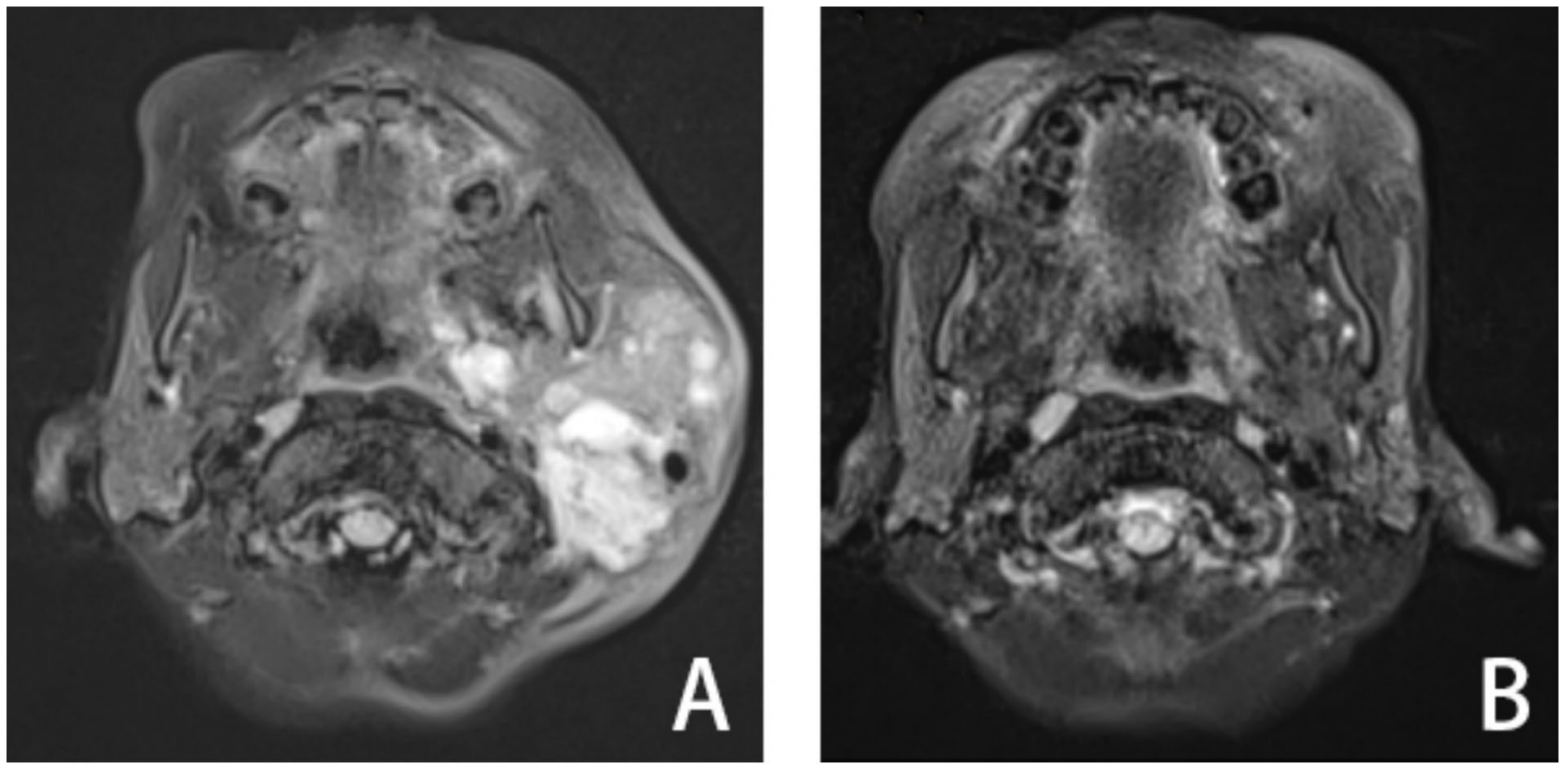
A patient with LM in maxillofacial region of T2-MRI axial scan: before treatment **(A)**, 1 year after treatment **(B)**.

**Figure 2:**
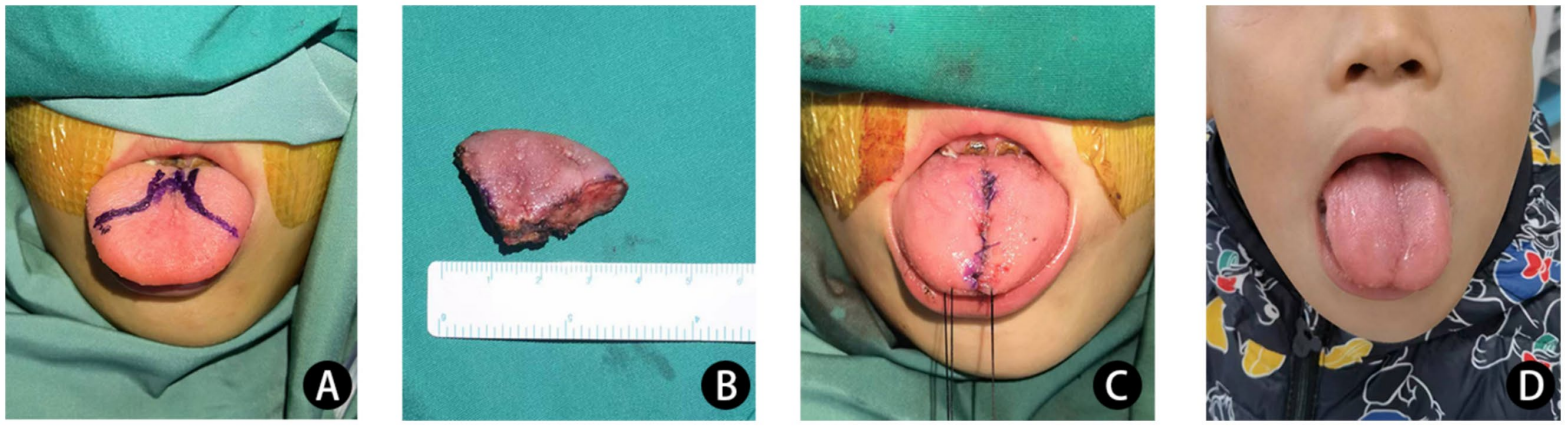
The patient had a large tongue, which affected mastication, swallowing and pronunciation, and there were lymphatic vesicles of different sizes on the surface of the tongue, some of which were accompanied by bleeding **(A)**. After partial removal of the tongue according to the marks, the tongue was thickened and tough **(B)**, and the tongue was reduced after surgery **(C)**. The 1-year postoperative follow-up showed improvement in mastication, swallowing and pronunciation **(D)**.

**Figure 3:**
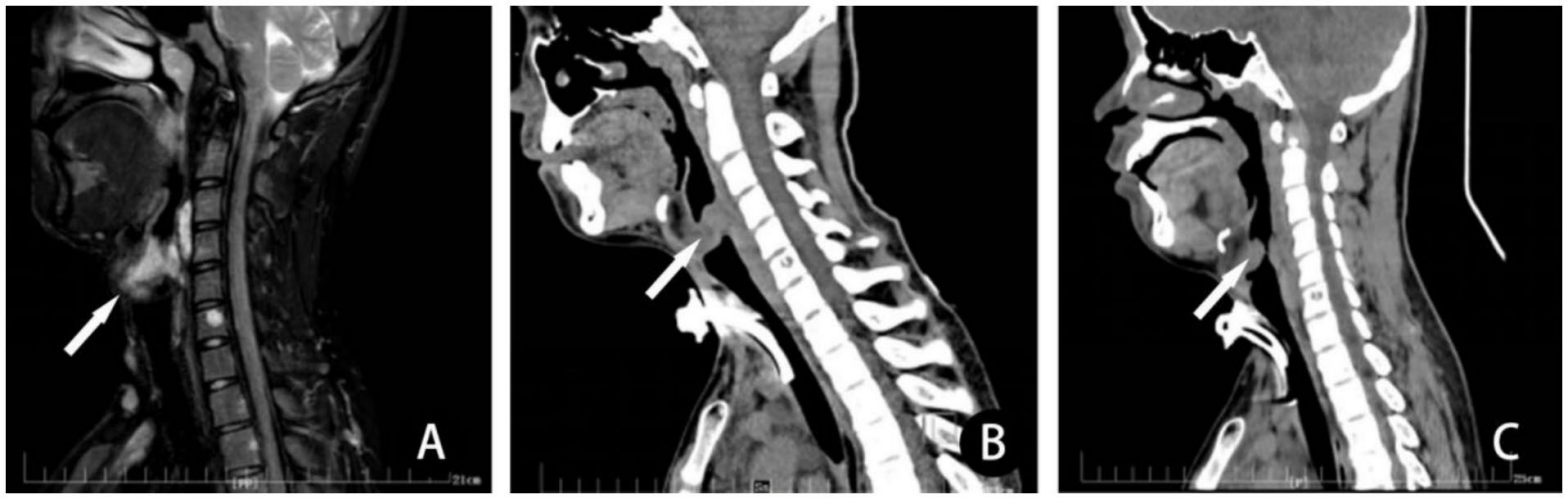
In a patient with LM involving the pharynx, **(A)** preoperative T2-MRI sagittal scan, revealed that the lesion involves and obstructs the airway (arrow) significantly. Three depressions sign was obvious when he walked fast. **(B)** He received a session of sclerotherapy with combination of ethanol and bleomycin after tracheotomy. Computerized tomography scan 1 month after treatment revealed severe obstruction of airway due to post-sclerotherapeutic reactive edema. **(C)** Computerized tomography scan 6 months after sclerotherapy demonstrated airway obstruction improved. Then, the tracheotomy tube was removed. There is a decrease in the size of the mass of the LM in the posterior subglottic LM lesion and little change in size of the mass in the anterior subglottic LM lesion. Despite this residual narrowing, the patient suffered no further sign or symptoms of airway obstruction.

Eighty-four patients underwent totally sclerotherapy and 10 underwent surgical resection. There were 39 females and 55 males, with an age ranging 1 to 11 years (median 2 years). Operative duration ranged 5–156 min (median 38 min). The follow-up period was 6 months to 1 year. The effect of sclerotherapy on 61 cases of large cystic LM in the head and neck was significantly improved, and the lesions were reduced by at least 50%, even >90% (Figure 1), without serious complications. In 5 patients with facial microcystic LM, for aesthetic considerations, parents preferred sclerotherapy to improve local enlarging, with poor short-term effect and only slight local improvement. In the long term, due to the stability of the lesions, the lesions may be relatively smaller with the growth and development of children. For patients with unsatisfactory effect of sclerotherapy, surgical treatment is combined with excision and sclerotherapy. Figure 1 shows the reduction of maxillofacial mixed cystic LM in a patient with a session of sclerotherapy. The therapeutic effect of a patient with a large microcystic lesion on the tongue is shown in Figure 2. Two patients had dyspnea due to pharyngeal involvement. One of the patients underwent endoscopic bleomycin injection and the other was treated with multiple sclerotherapy after tracheotomy (Figure 3), and the dyspnea was improved. There was only one complication in the excised patient, poor healing of the tongue wound resulted in a slight bifurcation of the tip of the tongue. The therapeutic effect was evaluated according to the size of the treatment area. There were 55 patients with excellent response, mainly sclerotherapy for macrocystic lesions and excision for localized microcystic or mixed lesions. A total of 21 patients were evaluated with good response, mainly with excision for macrocystic lesions and localized microcystic or mixed lesions which in order to protect skin blood transport without skin excess, and sclerotherapy for macrocystic or mixed lesions. There are 11 patients with moderate, mainly with sclerotherapy for microcystic and mixed lesions and excision for infiltrative microcystic or mixed lesions. There were 7 patients with no response, mainly sclerotherapy for microcystic or mixed lesions and excision therapy for microcystic lesions.

## Discussion

The clinical symptoms of lymphatic malformations on patients mainly depends on the location of the disease, the extent of the lesion and the type of cystic lesions. The lesions may present with bleeding, infection, enlargement, etc., resulting in varying degrees of dysfunction or affect appearance, such as megaloglossia, facial deformities, breathing, swallowing, articulation difficulties, etc. (5), requiring intervention and treatment. Among them, the anatomical structure of the head and neck is complex, there are many vital tissues and organs, and the treatment is more difficult with increased risks. LM in the head and neck are mainly cystic, and can be divided into macrocystic, microcystic and mixed types according to the size of the cystic cavity. There is no strict standard for the size of the cystic LM (6), but some authors use 2 cm (7) or 1 cm (8) as a limit to distinguish macro-versus micro-cystic, the significance of which is whether the cyst can be successfully aspirated to achieve visible decompression. The midface and oral lesions were mainly microcystic, the parotid gland and submandibular region were mainly mixed, and the neck was mainly macrocystic and mixed (9). Microcystic lesions located on the hyoid bone and on both sides are factors predicting difficult treatment and poor prognosis, especially the lesions involving the tongue, floor of the mouth and pharynx (3, 10). The goal of treatment is to remove LM as much as possible to control symptoms, restore function, or improve the appearance of the affected organ (11). At present, the main management methods include conservative observation, surgery, sclerotherapy, oral drug therapy and combination therapy. This article focuses on the comprehensive treatment of lymphatic malformations of head and neck.

At present, most scholars believe that not all LM needs treatment, that is, patients with mild appearance and no functional impairment can be followed up for observation (12). However, some scholars believe that LM is more likely to progress gradually, and the risk of progression of diffuse lesions is much higher than that of localized lesions, and early treatment is recommended (13). For less significant lesions, follow-up observation may be an option.

Surgical treatment has always been an important treatment for LM and is still widely used today (14). However, due to the complex anatomical structure of the head and neck, the aggregation of important organs, and the invasive growth of the lesions, there is no obvious boundary with the normal structure, which makes the operation difficult and the risk high, and the postoperative recurrence rate high, especially the microcystic lesions on the hyoid bone are more prone to recurrence (15). The lesions involved the mouth and face, and multiple anatomical sites (more than 2) suggested poor prognosis and high surgical complications (16). The principle of surgical treatment is to remove as many lesions as possible under the premise of protecting normal tissue and organ function. Surgical treatment is mostly used for microcystic and mixed LM. For macrocystic lesions, sclerotherapy can often achieve good results and is minimally invasive. However, it should be noted that sclerotherapy often cannot shrink the lesions in a short time. For serious emergency complications such as airway obstruction, surgery should be considered first. In recent years, compared with the traditional surgical treatment of LM, some new surgical techniques have emerged that deserve our attention and exploration, such as endoscopic resection (17), liposuction-like sclerotherapy technique (18), curettage and sclerotherapy technique (19), radiofrequency ablation (20), carbon dioxide laser (21), etc. It is important to note that the use of new techniques may cause severe swelling and may require a tracheotomy in advance.

In the past 20 years, sclerotherapy has gradually become another important method for the treatment of LM. At present, a variety of sclerosant have been clinically used, including OK-432, bleomycin, doxycycline, anhydrous ethanol, etc. (22). Double-needle technique is a safe and efficient technique (23). For larger macrocysts, suction drainage tube can be placed after sclerotherapy with multiple use of sclerosant (24). Most sclerosant have a good effect on large cystic lesions, followed by mixed lesions, and a limited effect on microcystic lesions (25). Anhydrous ethanol directly destroys endothelial cells, denatures proteins, and leads to thrombosis and fibrosis. The reasons for the use of anhydrous ethanol combined with bleomycin in the treatment of LM are as follows: (1) anhydrous ethanol can not only destroy lymphatic endothelial cells and cystic cavity, but also destroy small vessels that cause intracapsular bleeding, promote thrombosis and achieve hemostasis; (2) because the cyst cavity originally contained a large amount of lymph fluid, the irrigation process was a process of gradually increasing the concentration of ethanol, and the effect of destroying the cyst wall was better (3). The effect of anhydrous ethanol is strong but short, and the effect of bleomycin is mild but long lasting. The two complement each other to reduce the dose of bleomycin and reduce the side effects of pulmonary fibrosis. All kinds of sclerosant carry the risk of side effects, such as drug allergy, pulmonary fibrosis (unique to bleomycin), local pain, skin necrosis, nerve damage, generalized fever, skin pigmentation, and swelling (24). Compared with surgery, although sclerotherapy cannot remove the diseased tissue, it can still significantly reduce the lesions of some patients and even achieve the effect of “cure” on imaging, especially macrocystic lesions (26). Although there is also a high recurrence rate, the trauma ratio is significantly reduced by surgery, and the risk of nerve damage is lower than that of surgery.

At present, the oral drug commonly used in the non-surgical treatment of LM is sirolimus, which is an inhibitor of mTOR. It has achieved good results in the treatment of diffuse LM and is often used in patients with poor response to surgery and sclerotherapy. Although sirolimus cannot make the lesion disappear completely, it may improve the functional limitations, bleeding, pain, infection and exudation caused by the lesion (27). Oral ulcers, gastrointestinal discomfort, and bone marrow suppression are common side effects (28). Patients may need oral sirolimus lifelong, or have to be intermittently or permanently taken off sirolimus as complications and adverse events occur. Their symptoms will reappear when taking off sirolimus. In LM mouse models, both alpelisib and rapamycin improved mouse survival, but organ dysfunction was improved by only alpelisib, which is a p110α-specific PI3K inhibitor (29). This study implied that PI3K inhibitor may be superior to sirolimus in terms of LM treatment.

## Conclusion

Surgical resection, sclerotherapy and the combination of the two are efficacious treatment modalities for head and neck LM. Combined with oral drugs and other new therapies may be warranted in future for challenging conditions.

## Data Availability

The raw data supporting the conclusions of this article will be made available by the authors, without undue reservation.
